# Procedures for DNA Extraction from Opium Poppy (*Papaver somniferum* L.) and Poppy Seed-Containing Products

**DOI:** 10.3390/foods9101429

**Published:** 2020-10-09

**Authors:** Šarlota Kaňuková, Michaela Mrkvová, Daniel Mihálik, Ján Kraic

**Affiliations:** 1 Department of Biotechnology, Faculty of Natural Sciences, University of SS. Cyril and Methodius, Námestie J. Herdu 2, SK-917 01 Trnava, Slovakia; sarlota.kanukova@gmail.com (Š.K.); michaela.mrkvova@ucm.sk (M.M.); daniel.mihalik@ucm.sk (D.M.); 2 Research Institute of Plant Production, National Agricultural and Food Center, Bratislavská cesta 122, SK-921 68 Piešťany, Slovakia

**Keywords:** DNA extraction, opium poppy, seed, pollen grains, bakery product, oil, PCR

## Abstract

Several commonly used extraction procedures and commercial kits were compared for extraction of DNA from opium poppy (*Papaver somniferum* L.) seeds, ground seeds, pollen grains, poppy seed filling from a bakery product, and poppy oil. The newly developed extraction protocol was much simpler, reduced the cost and time required for DNA extraction from the native and ground seeds, and pollen grains. The quality of extracted DNA by newly developed protocol was better or comparable to the most efficient ones. After being extended by a simple purification step on a silica membrane column, the newly developed protocol was also very effective in extracting of poppy DNA from poppy seed filling. DNA extracted from this poppy matrix was amplifiable by PCR analysis. DNA extracted from cold-pressed poppy oil and suitable for amplifications was obtained only by methods developed previously for olive oil. Extracted poppy DNA from all tested matrices was analysed by PCR using primers flanking a microsatellite locus (156 bp) and two different fragments of the reference tubulin gene (553 bp and 96 bp). The long fragment of the reference gene was amplified in DNA extracted from native seeds, ground seeds, and pollen grains. Poppy DNA extracted from the filling of bakery product was confirmed only by amplification of short fragments (96 bp and 156 bp). DNA extracted from cold-pressed poppy oil was determined also only by amplification of these two short fragments.

## 1. Introduction

Extraction of nucleic acids from various matrices is the first and crucial step in analysis of biological materials generally. Methods of DNA extraction have evolved over time [1], but still contain several basic and necessary steps such as cell disruption, removal of undesirable molecules (lipids, proteins, polyphenols, and others), and purification. In addition to the cell wall disruption, the chemical diversity of metabolites contained in the plant cell is a major complication in the DNA isolation process. There is no available universal protocol for extraction of DNA which would be applicable independently of plant species, plant tissues, and plant matrix [2,3]. Generally, extraction of DNA from young, fast-growing, and healthy tissues is much easier. However, it is often necessary to extract DNA from plant tissues rich in polysaccharides, lipids, secondary metabolites, or even from very complex matrices (processed seeds, oils, foods, feeds). This is also the case of oilseeds where extraction of DNA is considered more demanding than from vegetative plant tissues (e.g., young leaves). Lipids usually prevent the action of solvents during removal of polysaccharides and phenolic compounds. Secondary metabolites can bind and precipitate with DNA and reduce efficiency of isolation procedure. Nevertheless, extraction of DNA from mature seeds may be often preferred over extraction from foliar tissues. Moreover, processing of plant seeds into foods is associated with determination of authenticity and traceability of foods what have recently become very important for various reasons [4,5,6]. The quantity and quality of DNA extracted from foods and oils tends to decrease to the extent in which the food/oil is processed [7,8]. Processing affects the DNA and may lead to degradation or removal of DNA from sample due to its hydrolysis, oxidation, and deamination [9]. Considering the DNA degradation and the presence of PCR inhibitors, DNA extraction from processed matrices is often a compromise between high yield and high purity [9,10,11]. The most appropriate extraction method should be chosen case by case. Extracted DNA is used for authentication of foods and feeds and detection of falsifications (e.g., blending of low-quality oil into high-quality oil) [12,13,14].

Oilseed crop with an interesting position in the world agriculture is the opium poppy (*Papaver somniferum* L.) grown under control only in some countries [15]. In addition to the production of alkaloids extracted from poppy straw, edible seeds are in great demand in cuisine. However, trading with poppy seeds, products (cake fillings, spreads), and oils suffers sometimes from adulteration practices [16]. Sometimes, high quality poppy seeds with a blue colour and a sweet taste are adulterated with technical poppy seed (grey-black colour, no taste). In addition to quality, they differ significantly in price. The consumer may be deceived in both quality and price. Such practices are then transferred to the food industry (poppy bakery products). Falsification is also a serious problem in the production of vegetable oils, especially the more expensive ones, including poppy seed oil. Chemical analyses of oils are used to determine the species origin of oil [17], but DNA analyses are appropriate to determine species origin and also the cultivar origin [18,19,20].

Poppy seeds with a high content of lipids and secondary metabolites are not a simple object for DNA extraction. This is even more complicated with ground seeds, poppy seed fillings from bakery products, and pressed oil. The number of relevant scientific reports in poppy is very limited and DNA extraction procedures have been published only from defatted seeds [21] and heroin samples [22]. Three commercial kits were tested for DNA extraction from seeds [23]. Very useful would be efficient, simple, and universal protocol for extraction of DNA from poppy seeds, grains, and products containing or made from poppy seeds. Therefore, the aim of this study was to test several methods of DNA extraction and try to design a new, effective procedure from different poppy seed matrices (native and ground seeds, pollen grains, poppy filling of the bakery product, poppy oil) with respect to DNA quality and suitability for amplification analyses.

## 2. Materials and Methods

### 2.1. Plant and Food Material

Mature seeds and pollen grains of opium poppy (*Papaver somniferum* L.) were collected from registered cultivar Major, cultivated at the Research and Breeding Station in Malý Šariš (Slovakia). They were stored at 4 °C before DNA extraction. Seeds and pollen grains were homogenized by pestle and mortar before the extraction. Seeds were also ground with the poppy seed mill. The poppy seeds roll (Tastino, Slovakia) and cold-pressed poppy seed oil (Juvamed Ltd., Tastino, Slovakia) were purchased in food store and stored at 4 °C before the DNA extraction.

### 2.2. DNA Extraction from Seeds

Genomic DNA from seeds was extracted from 0.2–0.5 g of seeds by six methods: Dellaporta et al. [24] with and without CTAB; Bayer BioScience N.V. [25]; Monsanto Company [26]; Murray and Thompson [27]; Sagwan et al. [21] and using four commercial kits: DNeasy^®^ Plant Maxi Kit, QIAamp DNA Stool Mini Kit, PowerSoil DNA Isolation Kit (all from QIAGEN N.V., Hilden, Germany) and Plant DNAzol^®^ Reagent (Thermo Fisher Scientific, Waltham, MA, USA).

Another extraction protocol was newly developed protocol designed on the basis of the Bayer BioScience N.V. procedure [25], but containing several modifications. The content of this protocol is as follows. The sample (200 mg) of seeds was ground to a fine powder with mortar and pestle and extracted with 2.7 mL of extraction buffer (50 mM EDTA, 100 mM Tris-HCl, pH 8.0, 500 mM NaCl), 190 μL of 20% SDS, and 10 μL of 2-mercaptoethanol. The mixture was vortexed and incubated at 65 °C for 30 min. During the incubation, the samples were mixed every 10 min. After incubation, 2.3 mL of mixture phenol:chloroform:isoamyl alcohol (25:24:1) was added, the mixture being shaken for 1 min and centrifuged for 20 min at 5500× *g*. The upper aqueous phase was transferred to a new tube, mixed with 2 mL of isopropanol and precipitated 30 min at −20 °C. Precipitated nucleic acids were transferred to Eppendorf tube and washed with 70% and 96% ethanol. Pellet after drying was dissolved in TE buffer (10 mM Tris-HCl, 1 mM EDTA, pH 8.0) and treated with 10 μL of RNase A (10 mg/mL) for 30 min at 37 °C. After the incubation, 800 μL of mixture chloroform:isoamyl alcohol (24:1) was added, shaken vigorously and centrifuged for 10 min in a microcentrifuge at maximum speed. The upper aqueous phase was transferred to a new Eppendorf tube, 600 μL of isopropanol was added and after vortexing was incubated for 20 min at −20 °C. Precipitate DNA was again washed with 70 and 96% ethanol, dried, dissolved in TE buffer, and stored at −20 °C.

### 2.3. DNA Extraction from Ground Seeds

Six different methods used for isolation DNA from 0.2 g of ground seeds were: Bayer BioScience N.V. [25], Monsanto Company [26], two commercial kits (DNeasy^®^ Plant Maxi Kit, QIAamp DNA Stool Mini Kit), and newly developed protocol (described above).

### 2.4. DNA Extraction from Pollen Grains

Three methods used for isolation of DNA from 0.1 g of pollen grains included DNeasy^®^ Plant Maxi Kit, QIAamp DNA Stool Mini Kit. The third was the newly developed protocol (described above). An efficient mechanical homogenization of pollen grains was particularly important.

### 2.5. DNA Extraction from Poppy Seed Filling

DNA was extracted from 0.5–2.0 g of filling of the bakery product using methods: Bayer BioScience N.V. [25], Monsanto Company [26], QIAamp DNA Stool Mini Kit, and newly developed protocol. Extracted DNA was purified through the silica membrane spin-columns [28].

### 2.6. DNA Extraction from Poppy Oil

DNA was extracted from 0.2–15 mL of oil according to Doveri et al. [29]; Monsanto Company [26]; Bayer BioScience N.V. [25]; Consolandi et al. [30]; Giménez et al. [31]; Raieta et al. [4], newly developed protocol, and commercial kit (QIAamp DNA Stool Mini Kit).

### 2.7. Qualitative and Quantitative Analysis of Extracted DNA

Integrity of the extracted DNA from different poppy matrices was assessed by agarose gel electrophoresis. Parameters of extracted DNA were tested by UV spectrophotometry (NanoDrop ND-1000 spectrophotometer, Thermo Fisher Scientific, Waltham, MA, USA) as well as by electrophoresis in 0.8% agarose gel stained with ethidium bromide.

### 2.8. PCR Amplification

Extracted DNA were amplified by PCR using primers for microsatellite locus psSSR69 [32]. Two pairs of primers for reference gene encoding tubulin beta-7 chain (Table 1) was designed from coding sequence (XM_026557633.1, GenBank^®^, http://www.ncbi.nlm.nih.gov) [33] using the Primer3 Input software (Whitehead Institute for Biomedical Research, USA).

PCR reactions were carried out in 15 μL reaction containing 11.7 μL ddH_2_O, 1.5 μL 10× PCR buffer, 0.3 μL of both primer (0.20 μM), 0.3 μL each of dNTP (200 μM), 0.2 μL Taq-polymerase (1U/μL), and 1 μL DNA (25 ng/μL). Parameters of PCR for the psSSR69 locus were: 94 °C for 3 min, 45 cycles of 45 s at 94 °C, 1 min at 54 °C, 1 min at 72 °C, and additional 1 cycle at 72 °C for 10 min. The reference gene for tubulin beta-7 chain was amplified using the program: 94 °C for 5 min, 35 cycles of 45 s at 94 °C, 1 min at 59 °C, 1 min at 72 °C, and additional 1 cycle at 72 °C for 5 min. Amplicons were analysed in 2% agarose gels in TBE buffer and stained with ethidium bromide.

## 3. Results and Discussion

### 3.1. DNA from Mature Seeds

Poppy seeds are specific commercial commodity used in the food industry particularly in some regions of the world. However, the food quality and related price of seeds vary considerably for different *P. somniferum* L. cultivars. Unfortunately, it is likely that premium quality seeds (sweet taste, blue colour) of some poppy cultivars are intentionally handled while trading. They are usually exchanged with low-quality seeds or mixed with them, whether intentionally or not. Therefore, different protocols for extraction of total DNA from poppy seeds and poppy containing products were tested. DNA analysis should be used to determine poppy seed cultivar origin. In addition to the six extraction protocols and four commercial kits tested (Table 2), the modified extraction procedure (“newly developed protocol”) was proposed in this study. It is based on the results and experiences obtained during testing of ten extraction procedures.

**Figure 1:**
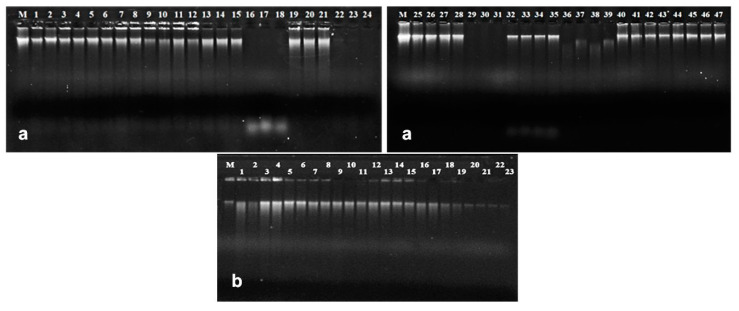
Genomic DNA extracted from opium poppy seeds (**a**) by: Dellaporta et al. [24] (lanes 1–6, where 1–3 homogenization with liquid nitrogen, 4–6 homogenization without liquid nitrogen), Dellaporta et al. [24] with CTAB (lanes 7–12, where 7–9 homogenization with liquid nitrogen, 10–12 homogenization without liquid nitrogen), Bayer BioScience N.V. [25] (lanes 13–15), Murray, Thompson [27] (lanes 16–18), Monsanto Company [26] (lanes 19–21), Sangwan et al. [21] (lanes 22–24), DNeasy^®^ Plant Maxi Kit (lanes 25–28), Plant DNAzol^®^ Reagent (lanes 29–31), QIAamp DNA Stool Mini Kit (lanes 32–35), PowerSoil DNA Isolation Kit (lanes 36–39), newly developed protocol (lanes 40–47). Lane M—λ-phage DNA. (**b**) Ground poppy seeds: DNA extracted by: Bayer BioScience N.V. [25] (lanes 1–7, lines 1–4, 0.2 g of seeds with extraction buffer volume for 0.2 g; lanes 5–7, 0.2 g of seed with extraction buffer volume for 0.5 g of seeds), Monsanto Company (26) (lanes 8–11), newly developed protocol (lanes 12–15), DNeasy^®^ Plant Maxi Kit (lanes 16–19), QIAamp DNA Stool Mini Kit (lanes 20–23). Lane M—λ-phage DNA.

Spectrophotometric analysis as well as gel electrophoresis of DNA from seeds revealed significant differences between used extraction protocols, both in quantity and quality of obtained DNA. The qualitative parameters of DNA were primarily important (Table 2). The protocol of Dellaporta et al. [24] and its modification by incorporation of CTAB showed that mechanical homogenization of seeds directly in the extraction buffer, even without the use of liquid nitrogen, did not lead to deterioration in quality or amount of DNA (Table 2, Figure 1a). It may be concluded that the need to use liquid nitrogen during mechanical homogenization of poppy seeds is not necessary for prevention of degradation of extracted DNA [34,35,36]. Quality of extracted DNA varied according to the extraction procedure. Procedures according to Sangwan et al. [21], Bayer BioScience N.V. [25], Monsanto Company [26], Murray and Thompson [27], QIAamp DNA Stool Mini Kit, DNeasy^®^ Plant Maxi Kit, as well as the newly developed protocol, provided poppy DNA with A_260/280_ values in range 1.77–2.11. Procedures Dellaporta et al. [24], PowerSoil DNA Isolation Kit, and Plant DNazol^®^ Reagent had the A_260/280_ ratios in range 1.54–1.75 (Table 2).

However, the success in amplification of extracted DNA is not guaranteed only by purity, but also by concentration and structural integrity of DNA [37,38]. Although values A_260_ of DNA extracted by protocols Murray and Thomson [27], Sangwan et al. [21], Plant DNAzol^®^ Reagent, and PowerSoil DNA Isolation Kit were high, DNA was not observed in agarose gel (Figure 1a, Table 2). There were probably only limited amounts of poppy DNA and absorbance values have been increased by the presence of RNA and other contaminants. DNA extracted by these protocols also had very low quality. Significant RNA contamination was reported only for the original CTAB method [27] and the QIAamp DNA Stool Mini Kit due to absence of RNase A treatment (Figure 1a).

**Figure 2:**
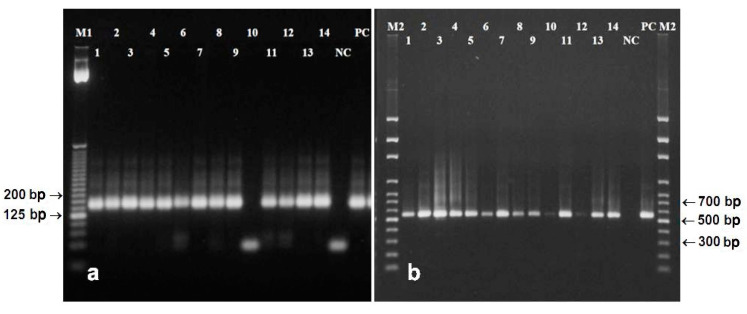
Amplification of 156 bp microsatellite psSSR69 (**a**) and 553 bp fragment of gene for tubulin beta-7 chain (**b**) in DNA extracted from poppy seeds by: Dellaporta et al. [24] with and without liquid N_2_ (lanes 1 and 2), Dellaporta et al. [24] with CTAB with and without liquid N_2_ (lanes 3 and 4), Bayer BioScience N.V. [25] (5), Murray, Thompson [27] (6), Monsanto Company [26] (7), Sangwan et al. [21] (8), DNeasy^®^ Plant Maxi Kit (9), Plant DNAzol^®^ Reagent (10), QIAamp DNA Stool Mini Kit (11), PowerSoil DNA Isolation Kit (12), newly developed protocol (13–14), NC—negative control, PC—positive control, M1—25 bp ladder (Invitrogen), M2—100 bp DNA ladder (Solis BioDyne).

Amplifications were successful from DNA extracted from mature native seeds by almost all of used protocols and the relevant fragments were generated (Figure 2). The only exception was DNA extracted by the Plant DNAzol^®^ Reagent. DNA extracted by PowerSoil DNA Isolation Kit was probably highly degraded considering that 553 bp fragment of reference tubulin gene was not amplified, but a 156 bp length microsatellite marker was generated (Figure 2).

The only one currently available protocol developed for extraction of DNA from poppy seeds [21] did not provide high quality of DNA within this study (Table 2, Figure 1a). The newly developed protocol has been proven as effective. Compared to the original protocol [25], extraction steps were rearranged, time intervals between steps were changed, and some chemicals/enzymes were eliminated. Both absorbance parameters (A_260/280_ and A_260/230_) as well as electrophoretic profile of DNA predicted very good quality and quantity (Table 2, Figure 1a) that should be suitable for amplification by PCR (Figure 2).

### 3.2. DNA from Ground Seeds

Ground poppy seeds are commonly available in food stores. The sensory values (especially taste and smell) and related varietal origin of high-quality seeds may be easily masked in ground seeds by various additives, mainly by sugar. Analytical testing and confirmation of the poppy seeds varietal origin is necessary in such cases. DNA from ground poppy seeds was extracted by two protocols [25,26], two commercial kits, as well as newly developed protocol. The QIAamp DNA Stool Mini Kit and DNeasy^®^ Plant Maxi Kit produced DNA with the A_260/280_ and A_260/230_ ratios furthest from optimal values (Table 2). Both spectrophotometric ratios of DNA extracted by Monsanto Company [26] protocol indicated high contamination of DNA with proteins, organic solvents, and secondary metabolites, and also very low concentration (Table 2). The yield of DNA was significantly different between tested protocols, but at the same amount of loaded DNA (25 ng/μL) the electrophoretic profiles of all DNA samples were appropriate (Figure 1b). The highest quality and concentration of DNA has been extracted by protocols Bayer Biocience N.V. [25] with changed ratio of sample–extraction buffer (w/v) and the newly developed protocol (Table 2, Figure 1b). Both protocols contained SDS in extraction buffer. It is suggested that SDS-based DNA extractions could be more appropriate for oily plant matrices like ground poppy seeds. The SDS-containing method modified for ground raw soybean seeds had the highest yield of DNA in comparison with the CTAB method and two commercial kits [39]. A lower amount of DNA yielded the CTAB method also from soybean flour [40].

**Figure 3:**
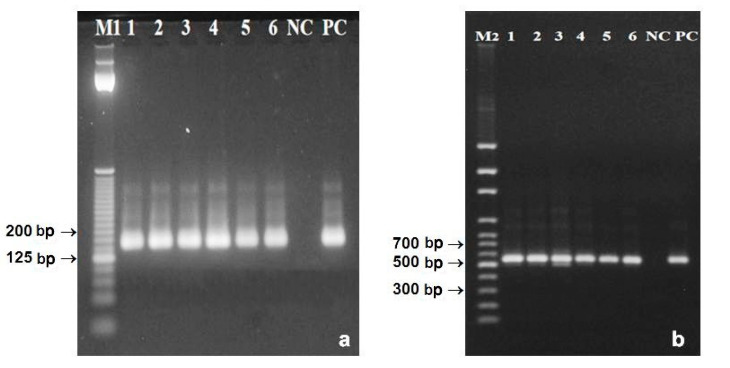
Amplification of 156 bp microsatellite psSSR69 (**a**) and 553 bp fragment of reference tubulin gene (**b**) in DNA extracted from ground seeds extracted by Bayer BioScience N.V. [25] (lanes 1–2, lane 1), Monsanto Company [26] (3), DNeasy^®^ Plant Maxi Kit (4), QIAamp DNA Stool Mini Kit (5), newly developed protocol (6), NC—negative control, PC—positive control, M1—25 bp DNA ladder (Invitrogen), M2—100 bp DNA ladder (Solis BioDyne).

Amplifications of DNA from ground poppy seeds using primers flanking microsatellite marker psSSR69 and longer fragment of gene for tubulin beta-7 chain resulted in production of both the 156 and 553 bp amplicons in DNA extracted by all used protocols (Figure 3).

### 3.3. DNA from Poppy Pollen Grains

**Figure 4:**
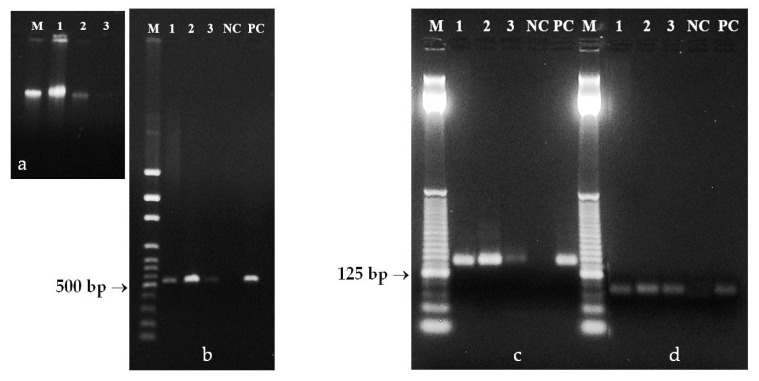
Genomic DNA extracted from opium poppy pollen grains (**a**). Amplification of 553 bp (**b**) and 96 bp (**d**) fragments of reference tubulin gene, and 156 bp (**c**) microsatellite, respectively. (1)—newly developed protocol, (2)—DNeasy^®^ Plant Maxi Kit, (3)—QIAamp DNA Stool Mini Kit, NC—negative control, PC—positive control, M—100 bp DNA ladder (**b**) (Invitrogen) and 25 bp DNA ladder (**c**,**d**) (Solis BioDyne).

DNA was extracted by two commercial kits and by newly developed protocol (Table 2). Homogenization by pestle and mortar in liquid nitrogen was efficient for disruption of pollen exine with high structural integrity. Both ratios A_260/280_ and A_260/230_ confirmed that the best quality had DNA extracted by newly developed protocol (Table 2). This simple protocol produced also very high amount of DNA. On the opposite, the QIAamp DNA Stool Mini Kit and DNeasy^®^ Plant Mini Kit extracted the least amount of DNA (Figure 4a). Amplifications of DNA from poppy pollen grains were basically without any complications. All primer pairs were able to amplify relevant amplicons (Figure 4). The genomic DNA is well protected inside the pollen grain therefore, a large fragment of the reference gene (553 bp) was simply amplified (Figure 4b). Amplifications of both shorter, the 156 bp microsatellite marker and 96 bp fragment of reference gene were also easily feasible (Figure 4c,d).

Extraction of DNA from pollen grains is needed in different applications including monitoring of pollen grains transfer from transgenic opium poppy plants to the environment [41], detection of pollen species in food (e.g., in honey) for the prevention of allergens [42], forensic palynology [43] and others.

### 3.4. DNA from Poppy Seed Filling

DNA was extracted by two procedures, one commercial kit, and the newly developed protocol (Table 3). The purification step using the silica membrane spin-columns [28] was added to protocols Monsanto Company [26] and newly developed one. Both ratios A_260/280_ and A_260/230_ confirmed that DNA extracted using almost all extraction protocols had these values out of the optimal range (Table 3). Undamaged high molecular weight DNA extracted from poppy seed filling from the bakery product was not visualizable in agarose gel (data not shown). This reflects fragmentation of poppy DNA to very short fragments due to high degradation during baking. This is common for DNA extracted from a matrix that has undergone processing by high temperature [29] and a combination of grinding, mechanical manipulation, and thermal treatment [44]. However, the objective quality and usability of DNA extracted can only be revealed by its amplification.

**Figure 5:**
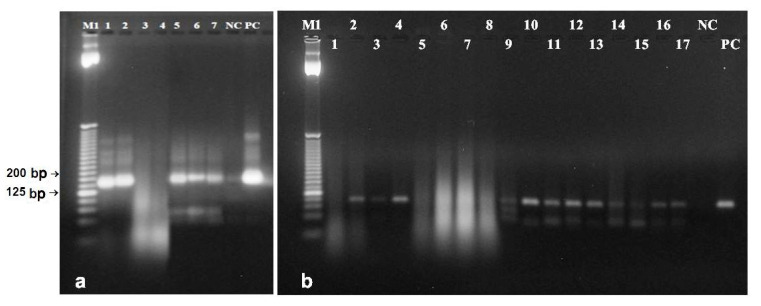
Amplification of 156 bp microsatellite psSSR69 in DNA extracted from the poppy seed filling (**a**) using: Bayer BioScience N.V. [25] (lane 1), QIAamp DNA Stool Mini Kit (2), newly developed protocol (3, 5, 7), Monsanto Company [26] (4, 6) NC—negative control, PC—positive control. Lanes 1–4 represent samples without, lanes 5–7 with purification through silica membrane columns. Amplification of 96 bp fragment of the reference tubulin gene (**b**) using: Bayer BioScience N.V. [25] (lanes 1, 2, 9, 10), QIAamp DNA Stool Mini Kit (lanes 3, 4, 11, 12), newly modified protocol (lanes 5–7, 13–16), Monsanto Company [26] (lane 8, 17) NC-negative control, PC-positive control. Lanes 1–8 represent samples without, lanes 9-17 samples with purification through columns. M1—25 bp DNA ladder (Invitrogen).

Complex food matrices contain a variety of PCR inhibitors [45]. Other effects of the matrix include degradation, fragmentation, and restricted extractability of DNA, as well as presence of DNA from different organisms [46]. Baking temperature around 200 °C used in processing of bakery goods containing poppy seed filling substantially reduces the size of extracted DNA. Moreover, higher moisture content inside the product, in this case in poppy filling, contributes to greater degradation of DNA [9]. Amplifications of poppy DNA extracted from filling of the baked product were more difficult. As expected, primer pair designed for amplification of 553 bp fragment of reference gene was not able to generate amplicon (data not shown). The Bayer BioScience N.V. method [25] and the QIAamp DNA Stool Mini Kit provided DNA with quality allowing amplification of the 156 bp microsatellite and short (96 bp) fragment of reference gene (Figure 5). Both these methods were effective also without the need of purification in columns. DNA extracted by the Monsanto Company method [26] and newly developed protocol was amplifiable only if the purification step in the silica membrane column [28] was added (Figure 5). Columns were able to bind impurities and inhibitors of polymerase chain reaction from primary DNA extracts.

### 3.5. DNA from Poppy Oil

**Figure 6:**
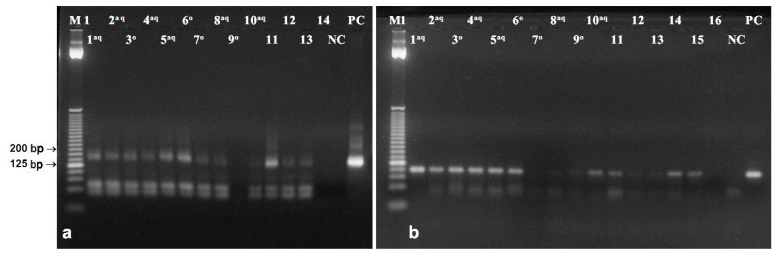
Agarose gel electrophoresis of PCR products obtained by amplification of 156 bp microsatellite psSSR69 (**a**) and 96 bp fragment of reference tubulin gene (**b**). M1—25 bp DNA ladder (Invitrogen). DNA extracted by Consolandi et al. [30] (lines 1–6) from 3 mL (1–3) or 6 mL (4–6) of oil, Raieta et al. [4] (lanes 7–10) from 3 mL (lanes 7,8) or 1 mL (lanes 9–10) oil, Doveri et al. [29] (line 11) extracted from 1 mL of oil, Giménez et al. [31] (lines 12–13) extracted from 0.5 mL (lane 12) or 3 mL (lane 13) of oil, QIAamp DNA Stool Mini Kit (lanes 14–16) extracted from 0.2 mL (lane 14b), 1 mL (lane 15b) or 15 mL (lanes 14a and 16b) of oil, NC—negative control, PC—positive control. ^aq/o^—DNA from water (^aq^) or oily (^o^) phase.

Oil from poppy seeds is mainly used for culinary and pharmaceutical purposes, but also for production of cosmetics, paints and varnishes. Cold-pressed oil is quite expensive, so it can sometimes be adulterated by much cheaper vegetable oils (e.g., from rapeseed, sunflower, oil palm). Techniques of analytical chemistry are developing for distinguishing between cheaper oils (e.g., sunflower, oilseed rape) and poppy oil [17]. However, chemical analysis may not be unambiguous [31] due to variation in chemical composition of vegetable oils among growing areas and seasons. Alternative approaches are based on the DNA analysis and require extraction of DNA from oil. Such protocols were developed mainly for olive oil. Four of such methods [4,29,30,31], the QIAamp DNA Stool Mini Kit as well as Bayer BioScience N.V. [25], Monsanto Company [26], newly developed protocols were tested for different volumes of poppy seed oil. Bayer BioScience N.V. [25], Monsanto Company [26] and newly developed protocol were unable to extract detectable and usable DNA (data not shown). DNA extracted by other protocols had also both absorbance parameters (A_260/280_, A_260/230_) far from the optimal values (Table 3); however, DNA was amplifiable by PCR (Figure 6). DNA in cold-pressed vegetable oil has undergone a process of significant degradation, caused by DNA nucleases released during crushing and malaxation of oily plant material. This will certainly happen when pressing oil from poppy seeds as well. If enzymatic mixtures of proteases are applied during this process, the DNA is prevented to damage and could be extracted with high integrity and concentration, similarly as from vegetative tissues [47]. However, this cannot be ensured in the already pressed oil. Another significant complication in the extraction of DNA is the time since pressing and conditions of the oil storage before the DNA extraction. After a relatively short time interval, a significant decreasing of quality of extracted DNA was observed due to oxidation damage [48]. Following the assumed high degradation, DNA has not even been electrophoretically controlled and only its amplifications revealed the potential utility of the extracted DNA. Statistical analysis did not reveal relationship between concentration, A_260_/A_280_ ratio, and the ability to undergo amplification by PCR [49].

Four extraction protocols [4,29,30,31] and the QIAamp DNA Stool Mini Kit provided different results (Figure 6). In addition, DNA extraction was also tested from different starting volumes of poppy seed oil. Extraction protocol developed for authentication of olive oils [30] was efficient either from 3 mL or 6 mL samples of poppy oil. Poppy DNA obtained by this protocol, from both the oily and water phases were amplifiable and provided templates for relevant amplicons. Other used DNA extraction protocols were also developed for olive oil, but based on the CTAB in extraction buffer [4,31]. The resulting poppy DNA behaved unreliably in the PCR reaction. Convincing and reliable amplifications were obtained from DNA extracted by another protocol, modified for olive oil [29] containing guanidine thiocyanate in extraction buffer. The capability of tested QIAamp DNA Stool Mini Kit for DNA extraction from poppy oil has been demonstrated in low oil volumes (0.2–1 mL).

The quality and quantity of DNA extracted from native or processed poppy seeds strongly depended on the character of poppy matrix entering the extraction procedure as well as level of its processing. Amplifications of obtained DNA were also influenced by many factors, especially by the presence of contaminants and inhibitors. Positioning of used primers for PCR analysis considered the expected length of extracted DNA fragments depended on the expected disruption of DNA during processing (baking, pressing) of poppy seed matrix. DNA extracted from different poppy seed matrices by different extraction protocols was amplified using primer pairs flanking the 553-, 156-, and 96 bp fragments, respectively (Table 1). The presence of the longest 553 bp fragment was detected by PCR in poppy DNA extracted from native seeds and ground seeds, but not from processed poppy seed matrices (filling of the bakery product, oil). Both types of poppy seed processing (baking, pressing) reduced the effective concentration of poppy DNA fragments capable of amplification of fragments longer than 100 bp, as was detected in maize cornmeal [50]. DNA from heat-processed and other highly degraded plant matrices should be amplified only in short DNA sequences. This is the strategy also in analysis of DNA from genetically modified organisms in processed foods [9,51]. Analysis of highly degraded DNA by PCR is more advantageous in DNA regions higher in GC content because their stability during heat treatment of the analysed matrices is higher [51].

Specific morphological characteristics, extreme heterogeneity and variation in chemical composition of plant cells cause many problems in DNA extraction. Although numerous protocols for plant DNA extraction have been published, none is found to be universally applicable [52]. Newly developed DNA extraction protocols are usually modifications of already existing protocols. The extraction protocol developed in our study demonstrated a relatively high degree of universality, with respect to poppy matrices. Compared to other DNA extraction protocols, it was quite universal. In comparison with the Bayer BioScience N.V. [25] protocol, from which the most steps were taken, it was approximately one third shorter in time. A significant reduction in time was achieved by adjusting the centrifugation steps. 2-mercaptoethanol ME was added to the first extraction buffer. Some steps during the extraction procedure were eliminated. Along with purification on silica membrane columns, the newly developed extraction protocol was highly efficient and represents a simple and inexpensive alternative to commercial DNA extraction kits. Extraction of DNA from oil required specific extraction protocols that were developed specifically for this type of matrix only.

## 4. Conclusions

Protocols tested for extraction of DNA from native and ground poppy seeds, pollen grains, poppy seed filling from the bakery product, and poppy oil have been differently effective and suitable depending on individual poppy seed matrices or products. DNA from seeds, ground seeds and pollen grains extracted by almost all extraction procedures had quantity and quality sufficient for PCR analysis of short microsatellite marker (156 bp) and also long fragment of the reference gene (553 bp). The best of these protocols have been tested for DNA extraction from the poppy seed filling from the bakery product. It has been very useful to use silica membrane columns for purification of the extracted DNA. Purified DNA was then amplifiable. Poppy DNA extracted from thermally processed poppy seed filling from the baking product did not amplify long fragment (553 bp) of the reference gene. However, primers designed for amplification of shorter fragment of the reference gene (96 bp) as well as for the microsatellite marker (156 bp) provided the appropriate amplicons. The new extraction protocol developed within this study has proven to be universally applicable to poppy seeds, pollen, and poppy seed containing products. It can be used for various control purposes in poppy breeding programmes, production and distribution of elite poppy seeds for crop production, control of poppy seeds identity as an interesting market commodity, control of products containing poppy seeds during food production. Protocols tested for extraction of poppy DNA from cold-pressed poppy oil were originally developed or modified for olive oil. The most of them [29,30] were effective, and extracted DNA was amplified using primers for the microsatellite marker and the short fragment of the reference gene.

## Figures and Tables

**Table 1:** Primer pairs used for amplification of opium poppy DNA. Tm: Melting temperature.

Primer Name	Sequences of Primers	Tm (°C)	PCR Product Size (bp)
psSSR69-F	5′-ATAGATTTATTTTGGCCACCT-3′	54.6	156
psSSR69-R	5′-CACCTATTGATTGAGGATGAA-3	55.2	
tubulin beta-7 chain I-F	5′-CGTGGGTCACAGCAATACAG-3′	59.4	96
tubulin beta-7 chain I-R	5′-ATGCCTAGGATCAGCAGCAC-3′	59.4	
tubulin beta-7 chain II-F	5′-AATCGGTGCAAAGTTCTGG-3′	54.5	553
tubulin beta-7 chain II-R	5′-GTTCCCATCCCAGATCCTG-3′	58.8	

**Table 2:** Parameters of DNA extracted from opium poppy native seeds, ground seeds, and pollen grains by different extraction procedures and commercial kits.

*P. somniferum* L. Matrix	DNA Extraction Method	DNA Yield (μg)	DNA Concentration (ng/μL)	A_260/280_	A_260/230_	Target Locus	
						psSSR69	Tubulin II
Native seeds	Dellaporta et al. [24]	82.76 *	413.78 *	1.66 *	0.87 *	+	+
		90.68	453.39	1.62	0.84	+	+
	Dellaporta et al. [24] with CTAB	53.32 *	266.62 *	1.62 *	0.83 *	+	+
		73.19	365.94	1.55	0.81	+	+
	Bayer BioScience N.V. [25]	115.13	575.63	1.81	1.84	+	+
	Murray, Thompson [27]	289.84	483.07	2.10	2.37	+	+
	Monsanto Company [26]	15.52	62.06	1.77	2.21	+	+
	Sagwan et al. [21]	108.21	1082.14	1.96	2.51	+	+
	Newly developed protocol	248.30	703.85	1.91	2.19	+	+
	DNeasy^®^ Plant Maxi Kit	108.83	145.11	1.96	2.18	+	+
	Plant DNAzol^®^ Reagent	137.40	114.50	1.54	0.28	-	-
	QIAamp DNA Stool Mini Kit	172.68	863.39	2.11	2.32	+	+
	PowerSoil DNA Isolation Kit	1.40	13.97	1.75	0.72	+	-
Ground seeds	Bayer BioScience N.V. [25] (1)	18.43	92.17	1.79	1.12	+	+
	Bayer BioScience N.V. [25] (2)	110.01	550.06	1.90	2.21	+	+
	Monsanto Company [26]	1.35	5.38	1.76	1.86	+	+
	DNeasy^®^ Plant Maxi Kit	6.28	35.88	1.53	0.64	+	+
	QIAamp DNA Stool Mini Kit	37.96	189.79	2.13	2.25	+	+
	Newly modified protocol	175.11	437.77	1.90	2.19	+	+
Pollen grains	Newly developed protocol	210.7	2107.0	1.95	2.17	+	+
	DNeasy^®^ Plant Mini Kit	2.22	22.2	1.92	1.1	+	+
	QIAamp DNA Stool Mini Kit	3.24	16.2	2.15	1.47	+	+

*—Homogenization of seeds with liquid nitrogen; Bayer BioScience N.V. [25] (1)—0.2 g of seeds extracted in volume of extraction buffer for 0.2 g seeds; Bayer BioScience N.V. [25] (2)—DNA extracted from 0.2 g of seeds extracted in volume of extraction buffer for 0.5 g of seeds.

**Table 3:** Parameters of DNA extracted from poppy seed filling and poppy seed oil using different extraction procedures and commercial kits.

*P. somniferum* L. Matrix	DNA Extraction Method	Weight (g)/Volume (mL) of Sample	DNA Yield (μg)	DNA Concentration (ng/μL)	A_260/280_	A_260/230_	Target Locus	
							psSSR69	Tubulin I
Poppy seed filling	Bayer BioScience N.V. [25]	1	79.91	399.55	1.94	1.92	+	+
	QIAamp DNA Stool Mini Kit	0.5	7.16	35.82	1.96	0.90	+	+
	Monsanto Company [26]	1	241.26	1206.28	2	1.75	-	-
	Monsanto Company [26]	1	0.97 *	32.41 *	1.57 *	0.40 *	+	+
	Newly developed protocol	2	246.55	1232.75	2.04	1.84	-	-
	Newly developed protocol	2	0.81*	26.91 *	1.68 *	0.59 *	+	+
Poppy seed oil	Doveri et al. [29]	1	0.43	7.1	2.53	0.01	+	+
	Consolandi et al. [30] aq	3	3.02	60.3	1.62	0.52	+	+
	Consolandi et al. [30] aq	3	0.94	18.8	1.48	0.54	+	+
	Consolandi et al. [30] oil	3	0.14	2.8	1.44	0.56	+	+
	Consolandi et al. [30] aq	6	2.19	43.8	1.40	0.57	+	+
	Consolandi et al. [30] aq	6	1.20	23.9	1.36	0.45	+	+
	Consolandi et al. [30] oil	6	0.21	4.1	1.22	0.59	+	+
	Giménez et al. [31]	0.5	0.08	3.0	1.75	0.08	+	-
	Giménez et al. [31]	1.5	0.06	2.5	1.28	0.06	+	-
	Raieta et al. [4] oil	3	0.18	6.1	1.26	0.13	+	-
	Raieta et al. [4] aq	3	0.26	8.7	1.61	0.21	+	-
	Raieta et al. [4] oil	1	0.10	3.3	2.07	0.09	-	-
	Raieta et al. [4] aq	1	0.13	4.3	1.26	0.12	+	+
	QIAamp DNA Stool Mini Kit	0.2	1.05	20.9	1.84	0.34	-	+
	QIAamp DNA Stool Mini Kit	1	1.71	34.2	1.78	0.33	-	+
	QIAamp DNA Stool Mini Kit	15	1.83	36.6	1.96	0.09	-	-

Note: *—subsequent purification of extracted DNA through silica membrane spin-columns [28], aq/oil—DNA isolated from the water (aq) or oily (o) phase.
